# Time to surgery after proximal femur fracture in geriatric patients depends on hospital size and provided level of care: analysis of the Registry for Geriatric Trauma (ATR-DGU)

**DOI:** 10.1007/s00068-023-02246-4

**Published:** 2023-03-16

**Authors:** Johannes Gleich, Carl Neuerburg, Carsten Schoeneberg, Matthias Knobe, Wolfgang Böcker, Katherine Rascher, Evi Fleischhacker, Working Committee on Geriatric Trauma Registry of the German Trauma Society (DGU), Berlin, Germany

**Affiliations:** 1 Department of Orthopaedics and Trauma Surgery, Musculoskeletal University Center Munich (MUM), University Hospital, LMU Munich, Marchioninistr. 15, 81377 Munich, Germany; 2 Department of Orthopedic and Emergency Surgery, Alfried Krupp Klinikum, Essen, Germany; 3 Westmünsterland Hospital, Ahaus, Germany; 4 Medical Faculty, RWTH University Hospital Aachen, Aachen, Germany; 5 Medical Faculty, University of Zurich, Zurich, Switzerland; 6 AUC, Akademie der Unfallchirurgie GmbH, Cologne, Germany

**Keywords:** Hip fracture, Level of care, Hospital size, In-house mortality, Geriatric trauma care

## Abstract

**Purpose:**

Proximal femur fractures predominantly affect older patients and can mark a drastic turning point in their lives. To avoid complications and reduce mortality, expert associations recommend surgical treatment within 24–48 h after admission. Due to the high incidence, treatment is provided at a wide range of hospitals with different size and level of care, which may affect time to surgery.

**Methods:**

Data from 19,712 patients included from 2016 to 2019 in the Registry for Geriatric Trauma (ATR-DGU) were analyzed in terms of time to surgery, in-house mortality, mobilization on the first postoperative day, ambulation status on the 7th day after surgery, and initiation of osteoporosis therapy. Participating hospitals were grouped according to their classification as level I, II or III trauma centers. Also presence of additional injuries, intake and type of anticoagulants were considered. Linear and logistic regression analysis was performed to evaluate the influence of hospitals level of care on each item.

**Results:**

28.6% of patients were treated in level I, 37.7% in level II, and 33.7% in level III trauma centers. There was no significant difference in age, sex and ASA-score. Mean time to surgery was 19.2 h (IQR 9.0–29.8) in level I trauma centers and 16.8 h (IQR 6.5–24) in level II/III trauma centers (*p* < 0.001). Surgery in the first 24 h after admission was provided for 64.7% of level I and 75.0% of level II/III patients (*p* < 0.001). Treatment in hospitals with higher level of care and subsequent increased time to surgery showed no significant influence on in-house mortality (OR 0.90, 95%-CI 0.78–1.04), but negative effects on walking ability 7 days after surgery could be observed (OR 1.28, 95%-CI 1.18–1.38).

**Conclusion:**

In hospitals of larger size and higher level of care the time to surgery for patients with a proximal femur fracture was significantly higher than in smaller hospitals. No negative effects regarding in-house mortality, but for ambulation status during in-hospital stay could be observed. As the number of these patients will constantly increase, specific treatment capacities should be established regardless of the hospitals size.

## Introduction

Proximal femur fractures can mark a turning point for elderly patients, as they are associated with high complication and mortality rates in this population. Rapid restoration of mobility turned out as a key factor in preservation of independency and activities of daily living. Therefore, prompt surgical treatment with the goal of immediate full weight bearing is essential [1–3]. When surgery is performed more than 48 h after admission, worse outcome regarding mobilization and mobility as well as significantly increased mortality have been observed [4]. This has led many trauma societies to recommend surgical treatment within 24–48 h after hospital admission for elderly patients with a fracture of the proximal femur [5–8].

Thus, a higher rate of surgeries out of hours, i.e., on weekends and at night is required. Various studies evaluated if out-of-hours surgery is associated with inferior outcome. Fatigue, a less experienced surgeon on duty or lack of knowledge in instrument-handling of the operating room staff were considered as some of the reasons against surgeries outside regular working hours. Contrary to this assumption, no increased duration of surgery, complication rates or mortality were observed. Thus, out-of-hours surgical treatment of older patients suffering a proximal femur fracture can be considered safe and is recommended [5, 8, 9]. In Germany, a resolution of a federal committee (Gemeinsamer Bundesausschuss, GBA) was enacted at the beginning of 2021, which mandates that patients with a proximal femur fracture undergo surgery within 24 h after admission and imposes severe fines on hospitals for non-compliance [10].

Up to now, only a few studies have examined why proximal femur fracture patients experience delay in surgical care [7, 11, 12]. Lieten et al. concluded that most delays occur because patients are deemed medically not fit enough, followed by, i.e., the need to optimize blood coagulation in anticoagulated patients or waiting for additional examinations and treatment by other disciplines [13]. Some studies also mentioned limited surgical capacity of the hospital or scheduling of these patients with minor priority [7, 13].

Due to the high incidence of proximal femur fractures, treatment is performed at a wide variety of hospitals. As they provide different levels of care, patients are scheduled with different priority for surgical treatment. For example, most of level I trauma centers are located at hospitals of maximum care, which have highly specialized departments, like organ transplantation, stroke or intensive care units. In these hospitals, patients suffering a proximal femur fracture are often not prioritized for surgery, as vital threatening entities have to be prioritized.

This study evaluates whether size and provided level of care of a hospital have an impact on time to surgery, in-house mortality, length of stay, mobilization on first day after surgery and ambulation status during inpatient stay in older patients with a fracture of the proximal femur.

## Patients and methods

In 2016, the German Trauma Society (DGU) founded the Registry for Geriatric Trauma (ATR-DGU). All hospitals certified as AltersTraumaZentrum DGU^®^ (ATZ-DGU) are required to enter data of their patients in this multicenter database. About 100 hospitals in Germany, Austria and Switzerland are currently involved. The data of all patients aged 70 years and older, who suffer a fracture of the proximal femur requiring surgery are queried. Initial data collection consists of standardized questionnaires, that represent five phases of hospitalization: admission, preoperative phase, surgery, postoperative phase, discharge [14]. The questionnaires were developed according to the Fragility Fracture Network (FFN), taking into account experiences from the “National Hip Fracture Database” of England and Wales, and the “Australian and New Zealand Hip Fracture Registry”, to allow international comparison. Parameters such as walking ability before the accident, a pre-existing level of care, intake of anticoagulants and osteoporosis medication upon admission, a geriatric assessment (including the ISAR score, a six-item screening tool for elderly patients in the emergency department, collecting data about functional dependence, recent hospitalization, impaired memory and polypharmacy), and general information on age and sex of the patients are collected. Information on time to surgery, fracture configuration, surgical and anesthesia procedures and ASA-Classification (American Society of Anesthesiologists) display the surgical phase. For the postoperative phase, walking ability, initiation of an osteoporosis treatment and interdisciplinary treatment by a geriatrician during the first seven postoperative days are documented. Information about the discharge location is also collected (home, rehabilitation clinic, nursing home, etc.). At two follow-up points (7 and 120 days after surgery), walking ability, status of osteoporosis treatment, re-operation rate and patients’ whereabouts are assessed.

Participating hospitals have to meet various criteria for certification as ATZ-DGU: interdisciplinary treatment by trauma surgeons and geriatricians, ensured geriatric treatment frequency (ranging from consultation based at least twice a week to continuous collaborative treatment), standard operating procedures for surgical treatment, pain management, mobilization, delirium assessment, prevention/therapy of osteoporosis, assessment and discharge management and many more.

In Germany, hospitals are assigned to different care levels on the basis of hospital care plans, where various aspects such as the number of beds are taken into account. The distinction made in the present study between level I and level II/III trauma centers matches with the classification of hospitals in the TraumaRegister DGU^®^: After completion of a certification process by the TraumaNetzwerk DGU^®^, trauma centers classified as “level I” correspond to the highest level of care and level III trauma centers to the lowest. For this evaluation, patients were divided into two groups: Group 1 includes patients treated at level I trauma centers, group 2 those treated at level II or III trauma centers. Complete data sets entered into the Registry for Geriatric Trauma (ATR-DGU) between 2016 and 2019 were analyzed. Patients suffering a periprosthetic or peri-implant femoral fracture were excluded, as extensive and therefore time-consuming preoperative planning may be necessary. Presence of additional injuries, intake and type of anticoagulants were also considered, as they could influence time to surgery, too. For differentiation of intake of anticoagulants (dichotomized in “yes/no”), acetylsalicylic acid (AA) or other antiplatelet therapy (AT) was not regarded as anticoagulant, in contrast to direct oral anticoagulants (DOAC). This takes into account, that surgery is not delayed by intake of AA or AT in most of the hospitals, whereas DOAC may influence the latency until surgery.

**Table 1:** Baseline characteristics of elderly patients undergoing hip fracture surgery in level I and level II/III trauma centers

	Level I	Level II/III	*p* value
Total patients	5636	14,076	
Total hospitals	19	61	
Age [years]			
Median (IQR)	85 (80;89)	85 (80;89)	0.985
Sex			
Female	4039 (71.8%)	10,168 (72.5%)	0.346
ASA			
1	63 (1.1%)	151 (1.09%)	0.287
2	1199 (21.6%)	3172 (22.9%)	
3	3866 (69.6%)	9430 (68.2%)	
4	421 (7.6%)	1073 (7.8%)	
5	3 (0.1%)	11 (0.1%)	
Additional injuries			
No	5128 (91.3%)	12,769 (91.2%)	0.805
Yes	488 (8.7%)	1234 (8.8%)	
Anticoagulant drugs			
No	4334 (79.9%)	10,792 (79.6%)	**0.015**
Yes	1089 (20.1%)	2762 (20.4%)	
Walking ability pre-fracture			**< 0.001**
Without aids	2029 (38.4%)	4227 (32.3%)	
With one crutch/cane	572 (10.8%)	1707 (13.0%)	
With 2 crutches/walker	1652 (31.3%)	4344 (33.1%)	
Only at home	840 (15.9%)	2422 (18.5%)	
None	188 (3.6%)	405 (3.1%)	
Type of fracture			
Femoral neck	2373 (42.1%)	6615 (47.0%)	**< 0.001**
Pertrochanteric	2944 (52.2%)	6637 (47.2%)	
Subtrochanteric	215 (3.8%)	621 (4.4%)	
Other	104 (1.9%)	203 (1.4%)	
Type of surgery			
Screw osteosynthesis	48 (0.8%)	255 (1.8%)	
Dynamic hip screw	416 (7.3%)	269 (1.9%)	
Intramedullary nailing	2775 (49.0%)	7176 (50.6%)	
Hemiarthroplasty	1492 (26.4%)	5139 (36.2%)	
Total hip replacement	503 (8.9%)	1052 (7.4%)	
Other	426 (7.5%)	300 (2.1%)	
Hospital stay [days]	14.1 (10;22)	16 (10.1; 22)	**0.005**
Osteoporosis treatment pre-fracture			
Yes	1302 (24%)	2484 (18.3%)	**< 0.001**
Osteoporosis treatment 7 days after surgery			**< 0.001**
Yes	4062 (72.4%)	8771 (62.6%)	
Mobilization 1 day after surgery			**< 0.001**
Yes	3727 (67.1%)	11,589 (83.3%)	
Walking ability 7 days after surgery			**< 0.001**
No mobility	1304 (24.1%)	2670 (19.7%)	
Able to walk (with/without assistance)	4112 (75.9%)	10,854 (80.3%)	
Time to surgery [h]			**< 0.001**
Median (IQR)	19.2(9.0;29.8)	16.8 (6.5; 24)	
Time to surgery [categorical]			**< 0.001**
≤ 24 h	3626 (64.7%)	10,468 (75.0%)	
> 24 h	1978 (35.3%)	3497 (25.0%)	

Significant p values are in bold

Mann–Whitney *U* Test was used for continuous variables, chi-squared test for discrete variables

*ASA* American Society of Anesthesiologists

### Statistics

The infrastructure for data entry, data management and data analysis are provided and maintained by the AUC—Academy for Trauma Surgery (AUC), an institution affiliated with the German Trauma Society (DGU). The scientific leadership is incumbent on the Working Committee on Geriatric Trauma Registry (AK ATR) of the DGU. The scientific data analysis is approved according to a peer-review process defined in the ATR-DGU publication policy. The present study was approved with project number ATR-2020-008. Data analysis received approval from the Ethics Committee of the medical faculty of the LMU Munich, Munich, Germany (Reg. No. 234-16) and from the Ethics Committee of the medical faculty of the Philipps-University, Marburg, Germany (AZ 46/16). Data are available from the Registry for Geriatric Trauma (ATR-DGU) after approval by the Working Committee on Geriatric Trauma Registry (AK ATR) of the DGU. This study followed the Strengthening the Reporting of Observational Studies in Epidemiology (STROBE) reporting guideline for cohort studies.

For descriptive analyses, categorical data were presented as counts and percentages, continuous variables as median with interquartile range (IQR). Some patients had missing data for individual parameters; therefore, each analysis shows the total number of patients that were analyzed. Comparisons between the two groups were made using *Χ*^2^-test for categorical variables and the Mann–Whitney *U* Test for continuous variables. Linear and logistic regression models were used to examine the impact of the size of hospital and following time to surgery on a range of outcomes 7 days after surgery. All multivariate analyses were adjusted for age, gender and ASA score, additional injuries and anticoagulation before fracture. Results are reported as regression coefficient (*β*) for linear regression and Odds Ratios (OR) for logistic regression along with their 95%-confidence intervals (CI). Differences were considered statistically significant when *p* < 0.05. All calculations were performed using statistics software R v. 4.0.2 (Foundation for Statistical Computing, Vienna, Austria).

## Results

19,712 patients from 80 hospitals (19 level I and 61 level II/III trauma centers) were included for final analysis. Women represented 72% of the study population and median age was 85 (IQR 80–89) years. In 76.4% of the patients a severe systemic disease was observed (ASA ≥ 3); prior to trauma, 38.4% of level I and 32.3% of level II/III patients could walk unaided and nearly 80% of all patients had no existing osteoporosis treatment. 28.6% of patients were treated in a level I trauma center and 37.7%/33.7% of the patients in a level II/III trauma center. Baseline data showed no differences between the groups in age, gender, ASA score or the presence of additional injuries (Table 1). 20.1% (level I) and 20.4% (level II/III) of the patients had anticoagulants in their permanent medication. Median time to surgery was significantly longer in level I trauma centers (19.2 h, IQR 9.0–29.8) than in level II/III hospitals (16.8 h, IQR 6.5–24; *p* < 0.001). Surgery in the first 24 h after admission was provided for 64.7% of the patients at level I trauma centers and for 75.0% of the patients at level II/III trauma centers (*p* < 0.001). The most common surgical procedure was intramedullary nailing of a trochanteric (per-/intertrochanteric) fracture in both groups with 41.8% (level I) and 50.6% (level II/III) of the patients. Linear regression also revealed significantly longer time to surgery in level I trauma centers (*β* 4.81 h, CI 3.96–5.66) compared to level II/III hospitals (Table 2). Multivariate logistic regression analysis showed no significant influence of hospital size on in-house mortality (OR 0.90, CI 0.78–1.04), while walking ability 7 days after surgery was reduced in treatment in level I trauma centers (OR 1.28, CI 1.18–1.38).

**Table 2:** Multivariable linear and logistic regression analysis of the impact of treatment in a level I trauma center on various outcomes during hospital stay

Impact of treatment in a level I trauma center on…	*N*	*β*	95%-CI	*p* value
Time to surgery in hours^a^	19,019	4.81	[3.96; 5.66]	**< 0.001**
Hospital stay in days^a^	17,981	− 0.51	[− 0.80; − 0.23]	**< 0.001**

	*N*	OR		
Inhouse mortality^b^	19,130	0.90	[0.78; 1.04]	0.155
No walking ability 7 days after surgery^b^	18,426	1.28	[1.18; 1.38]	**< 0.001**

Significant p values are in bold

All models were adjusted for sex, age, ASA Score, additional injuries and pre-existing anticoagulant drugs

^a^Linear regression analysis

^b^Logistic regression analysis

Some observed significance differences (e.g., Walking ability pre-fracture or type of fracture) have to be interpreted with caution, as they are attributed to the large sample size and not to actual inter-group differences.

## Discussion

This evaluation of the Registry for Geriatric Trauma (ATR-DGU) showed significantly longer time from hospital admission to surgery for patients with a proximal femur fracture in level I trauma centers compared to level II/III trauma centers, with surgery in 64.7%/75.0% of patients performed within 24 h after admission. There are many possible reasons for delayed surgery, which can be divided into patient-associated and logistic problems. In the past, various studies already tried to assess this question. Large evaluations of patients with a proximal femur fracture from the US, Canada, Germany and most recently from Belgium have shown that in most cases medical, patient-related reasons were responsible for delay in the surgical treatment, as they were not fit enough for the procedure [13, 15–17]. A second important factor resulting in delayed surgery was impaired blood clotting due to the intake of anticoagulants was listed [13, 17]. However, this cannot explain the delay observed in level I trauma centers in this study, as there was no difference in anticoagulation status between the groups. Logistic reasons such as lack of operating room (OR) capacity and qualified staff are also possible reasons for delay. Yet, overall numbers are difficult to compare due to very heterogeneous inclusion criteria. Orosz et al. stated already in 2002 the overall percentage of these factors at 41% [15]. In a recent study by Lieten et al. 4.6% of OR delays were caused by a lack of OR capacity [13]; also relationship between hospital size and delay from admission to surgery in patients with a proximal femur fracture have already been investigated [18–20]. Kristensen et al. demonstrated that time from admission to surgery increased with hospital size in an evaluation of the Danish hip fracture registry [18]. This supports the results presented in this manuscript, as well as data from the Canadian Hip Fracture Registry [21]. Again, Weller et al. observed the longest time from admission to surgery at hospitals with the highest level of care [21]. In contrast to this, Metcalfe et al. and Elkassabany et al. demonstrated in data from US hip fracture patients, the smaller the hospital, the more surgery was delayed [19, 20]. Various differences between the US and Danish/German public health care systems might explain these findings. Also a German-wide survey in chairpersons of trauma departments from 2013 stated that 98% of participating hospitals performed surgery in patients with unstable intertrochanteric fractures within 24 h after admission and more direct surgeries were seen in level I hospitals [22]; comparability of these (contrary to the present) findings is limited, because only one specific fracture pattern was evaluated and data were collected on a survey basis, whereas data of this study were gathered by a large registry.

Hospital volume and in-house mortality presented no relation in this study, which corresponds to the varying data of previous evaluations: Kristensen et al. showed that admission to high-volume hospitals was associated with increased odds for dying within 30-days [18]. Contrary results were obtained in a study of US hip fracture data, where mortality was lower at (bigger) teaching hospitals compared to urban community hospitals [21]. Following an analysis of the present registry data by Schoeneberg et al., increased time to surgery, regardless of the providing hospitals level of care, showed no significant effect on in-house mortality [23].

Increased odds for worse walking ability 7 days after surgery were found in level I trauma centers. In addition, mobilization on the first day after surgery was performed significantly more often in level II/III trauma centers. Data of the Danish Hip Fracture Registry on mobilization of patients on the first postoperative day support these results [18]. This might be attributable to longer time to surgery in level I trauma centers, which could have delayed initial mobilization and therefore adversely affected subsequent ambulation status during inpatient stay. Compared to maximum care hospitals, in level II/III trauma centers more patients suffering a femoral neck fracture, which were treated by (hemi)arthroplasty, were observed, which might also have influenced mobilization and walking ability.

Regarding initiation of osteoporosis therapy, higher frequency was observed in level I trauma centers. These findings match with an evaluation of the Spanish Hip Fracture Registry by Alarcon et al., where anti-osteoporotic therapy was prescribed significantly more often at high-volume hospitals [24]. Furthermore, Kristensen et al. found that interventions to prevent future falls were taken more frequently at high-volume hospitals [18].

Strengths of this study are the large patient collective with prospective data collection and complete follow-up, which minimized the risk of selection and information bias. Patients with a proximal femur fractur in Germany are generally admitted to the closest hospital with a trauma unit and treatment capacity. Therefore, selection by health status, fracture severity, or other characteristics does not occur. This is supported by the data presented above, where no difference between the study groups in terms of ASA classification or additional injuries was demonstrated. The risk of confounding-by-indication was minimized by recording various preoperative factors such as functional level and pre-existing cognitive impairment as well as pre-existing need for care. Nevertheless, influence by unmeasured confounding is possible. Another limitation of this study relates to the reliability of the data, as they were collected from a large number of hospitals and physicians. To reduce heterogeneity, data collection is based on standardized questionnaires; moreover, all participating hospitals are certified as geriatric trauma centers and are assessed in a series of regular clinical audits. In addition, no information on complications caused by increased time to surgery, besides surgical ones, are available, as they are not queried.

## Conclusion

With regard to time from admission to surgery for patients with a proximal femur fracture older than 70 years, analysis of the Registry of Geriatric Traumatology (ATR-DGU) showed a significantly longer time in level I trauma centers compared to level II/III trauma centers, with 64.7% and 75.0% of patients undergoing surgery within 24 h after admission. There was no statistically significant difference in in-house mortality between hospitals with different levels of care, but better walking ability 7 days after treatment was observed in hospitals providing lower level of care, which also showed shorter time to surgery.
